# Protein Co-Aggregation Related to Amyloids: Methods of Investigation, Diversity, and Classification

**DOI:** 10.3390/ijms19082292

**Published:** 2018-08-04

**Authors:** Stanislav A. Bondarev, Kirill S. Antonets, Andrey V. Kajava, Anton A. Nizhnikov, Galina A. Zhouravleva

**Affiliations:** 1 Department of Genetics and Biotechnology, St. Petersburg State University, Universitetskaya nab., 7/9, St. Petersburg 199034, Russia; kirantonez@gmail.com; 2 Laboratory of Amyloid Biology, St. Petersburg State University, Russia, Universitetskaya nab., 7/9, St. Petersburg 199034, Russia; 3 Laboratory for Proteomics of Supra-Organismal Systems, All-Russia Research Institute for Agricultural Microbiology, Podbelskogo sh., 3, Pushkin, St. Petersburg 196608, Russia; 4 Centre de Recherche en Biologie cellulaire de Montpellier (CRBM), UMR 5237 CNRS, Université Montpellier 1919 Route de Mende, CEDEX 5, 34293 Montpellier, France; Andrey.Kajava@crbm.cnrs.fr; 5 Institut de Biologie Computationnelle (IBC), 34095 Montpellier, France; 6 University ITMO, Institute of Bioengineering, Kronverksky Pr. 49, St. Petersburg 197101, Russia

**Keywords:** amyloid, prion, co-aggregation, cross-seeding, neurodegenerative diseases, functional amyloids, RHIM

## Abstract

Amyloids are unbranched protein fibrils with a characteristic spatial structure. Although the amyloids were first described as protein deposits that are associated with the diseases, today it is becoming clear that these protein fibrils play multiple biological roles that are essential for different organisms, from archaea and bacteria to humans. The appearance of amyloid, first of all, causes changes in the intracellular quantity of the corresponding soluble protein(s), and at the same time the aggregate can include other proteins due to different molecular mechanisms. The co-aggregation may have different consequences even though usually this process leads to the depletion of a functional protein that may be associated with different diseases. The protein co-aggregation that is related to functional amyloids may mediate important biological processes and change of protein functions. In this review, we survey the known examples of the amyloid-related co-aggregation of proteins, discuss their pathogenic and functional roles, and analyze methods of their studies from bacteria and yeast to mammals. Such analysis allow for us to propose the following co-aggregation classes: (i) titration: deposition of soluble proteins on the amyloids formed by their functional partners, with such interactions mediated by a specific binding site; (ii) sequestration: interaction of amyloids with certain proteins lacking a specific binding site; (iii) axial co-aggregation of different proteins within the same amyloid fibril; and, (iv) lateral co-aggregation of amyloid fibrils, each formed by different proteins.

## 1. Introduction

Amyloids are unbranched protein fibrils, in which monomers form intermolecular β-structures stabilized by numerous hydrogen bonds and consisting of β-strands oriented perpendicular to the axis of the fibril (reviewed in [1,2,3]). This ordered spatial structure, called cross-β, is unusually highly resistant to treatment with proteases and ionic detergents, SDS (sodium dodecyl sulfate) and sarcosyl (sodium lauroyl sarcosinate), high temperatures, acids and alkalis (reviewed in [4]). Amyloids represent one of the most stable biogenic particles, with some of them preserving their properties in the external environment for years (reviewed in [5]). The specific structure of amyloids can be detected with different biophysical methods. Amyloids exhibit apple-green birefringence under the polarized light upon binding of Congo Red (CR) dye (the “gold standard” to prove the amyloid nature of the protein aggregates in clinical practice [4]) and to demonstrate so-called “cross-β pattern” in X-ray fiber diffraction. Also, amyloid fibrils can be detected by an increase in the fluorescence emission of the benzothiazole dye Thioflavin T (ThT). The other methods for investigating the amyloid structure include atomic force and electron microscopy, infrared spectroscopy, mutagenesis, and NMR (Nuclear Magnetic Resonance) (reviewed in [6]).

Initially, amyloids were discovered as macroscopic tissue abnormalities (accumulation of the abnormal, predominantly extracellular, protein deposits) linked with different diseases (reviewed in [1]). To date, more than 30 proteins have been demonstrated to be associated with various incurable diseases in humans and animals called amyloidoses. These proteins include Aβ (amyloid-β peptide) and Tau, both being associated with Alzheimer’s disease (AD), α-synuclein (α-Syn)—Lewy body disease (LBD) and Parkinson’s disease (PD), huntingtin protein (Htt)—Huntington disease (HD), islet amyloid polypeptide (IAPP)—diabetes type II, and prion protein (PrP)—Creutzfeldt–Jakob disease (CJD) (reviewed in [4]). On the other hand, numerous studies have demonstrated that amyloids are implicated in various biological processes in a wide spectrum of organisms. In prokaryotes, amyloids play different biological roles, including the formation of biofilms [7,8] and extracellular cell wall sheaths [9], “multicellular” growth [10], and sequestration of toxins [11,12]. In animals, functional amyloids participate in melanin polymerization, hormone storage, programmed necrosis, and long-term memory formation (reviewed in [13]).

According to classical definition, prions are infectious proteins (prion, from proteinaceous infectious particle) [14]. Most of the known prions (with only a few exceptions, for instance, [β] and Rho [15,16]) are associated with the formation of amyloid aggregates by corresponding prion protein. In this review, we will discuss only amyloid-forming prions. In humans and animals, there is only one known prion, PrP^Sc^ (Sc, from “scrapie”, the prion disease of sheeps; noninfectious cellular isoform of PrP is designated PrP^C^) [14]. At the same time a range of prions was found in lower eukaryotes. The well-known examples are [*PSI*^+^], [*PIN*^+^] and [URE3], which are associated with the amyloid aggregation of Sup35, Rnq1, or Ure2, respectively (reviewed in [17]). Increasing evidence shows that some of the disease-associated amyloid proteins may have prion-like properties (reviewed in [18]). The pathological transmission of misfolded proteins by prion-like mechanisms was demonstrated for several neurodegenerative diseases [19,20], and the existence of a special cell-to-cell propagation mechanism for prion-like proteins was proposed (reviewed in [21]). While in humans and animals the discovered prion and prion-like proteins are lethal pathogens causing neurodegenerative diseases, in fungi prions may be both lethal and functional (reviewed in [22]). Overall, amyloids and prions may play distinct roles either representing the pathogenic protein misfolding or encompassing the special protein structure that is essential for different cellular processes.

Recent studies have demonstrated that formation of amyloid fibrils not only involves aggregation of the particular protein, but also has a significant influence on the quantity of some other proteins and the depletion of their functions caused by co-aggregation. For example, it was demonstrated that α-Syn might serve as a promiscuous binder, leading to its co-aggregation with other proteins or modulation of their activities (reviewed in [23]). The analysis of known experimental data made in this review allows for us to propose the following co-aggregation classes: (i) titration: deposition of soluble proteins on the amyloids formed by their functional partners, with such interactions mediated by a specific binding site; (ii) sequestration: interaction of amyloids with certain proteins lacking a specific binding site; (iii) axial co-aggregation of different proteins within the same amyloid fibril; and, (iv) lateral co-aggregation of amyloid fibrils, each formed by different proteins. This network of interactions between amyloids and other proteins (that can be called the amyloid interactome) may have significant pathological and functional impacts. In this review, we examine methodology for studying amyloid-related interactions, summarize the rapidly growing data on the interactions of amyloids with other proteins, discuss their biological significance, and propose the classification of these interactions.

## 2. Methods for Investigation of the Amyloid Interactome

Traditional methods for investigation of protein-protein interactions can also be used to characterize interactions either between different amyloids or between monomeric proteins and amyloids. These methods include co-immunoprecipitation, colocalization, affinity chromatography, gel filtration, and other techniques. Also, several amyloid specific techniques have been developed. Among them, are cross-seeding, co-incubation, and other methods, including proteomics and bioinformatics approaches. Typically, several methods are combined to characterize the interaction of specific proteins with amyloid aggregates. Some of these methods are discussed below. Similar approaches are grouped together.

### 2.1. Co-Immunoprecipitation

**Figure 1:**
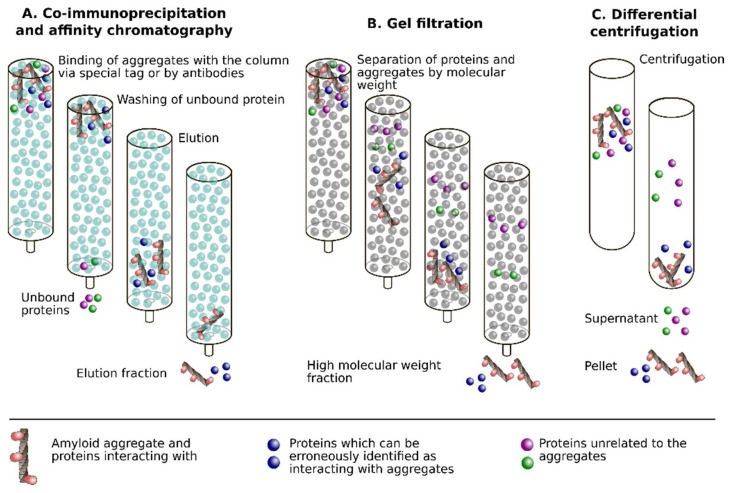
Schematic representation of approaches for identification of proteins co-aggregating with amyloids: co-immunoprecipitation or affinity chromatography (**A**), gel filtration (**B**) and differential centrifugation (**C**). In all approaches, some proteins can be erroneously identified as interacting with amyloids due to different reasons, listed below. In the case of co-immunoprecipitation (co-IP) or affinity chromatography, it is non-specific interaction with antibodies or with the affinity chromatography resin. The high molecular weight of the protein or its inclusion in various complexes may lead to the misleading assumption that the protein interacts with amyloids. Finally, independently on technique, the excess of the protein in the sample may cause erroneous results.

Co-immunoprecipitation (co-IP) is a widely used assay to study protein interaction in vivo. This approach includes the extraction of a certain protein from the lysate or in vitro prepared mixture of proteins with a specific antibody and subsequent identification of co-eluted proteins (Figure 1A). For example, using a monoclonal antibody specific for Aβ, co-precipitation of Aβ, and PrP in brain homogenates was shown [24]. Reciprocal co-IP assays have documented the interaction of α-Syn with hyperphosphorylated Tau in solubilized lysates from mouse neurons that are treated with the Parkinsonism-inducing neurotoxin MPP^+^ [25]. The same approach was used to prove the interaction among Aβ and α-Syn in the cases of LBD and AD. In these studies, reciprocal co-IP assays have shown the strongest interaction between Aβ and α-Syn in the LBD cases, less strong in the AD samples, but none in control non-demented samples [26]. The major limitation of this approach is its inability to provide information whether the proteins are incorporated in or only bind the aggregate.

### 2.2. Affinity Chromatography

Affinity chromatography is one of the widely used methods allowing for one to purify specific molecules interacting with protein bound to the affinity matrix (Figure 1A). In general, this approach is very similar to co-IP. These methods differ only in the mechanism of bait protein (aggregate) immobilization on the column. Affinity chromatography requires fusion of the investigated protein with a specific tag. A detailed methodology to identify amyloid-binding proteins by using affinity chromatography has been recently described [27]. This approach was used to find α-Syn binding proteins in human brain cytosol preparations and allowed for finding human brain Tau as an α-Syn ligand [28]. In the yeast system, the presence of Rnq1 in the Sup35 aggregates was shown by the isolation of Sup35NM (amyloidogenic fragment of the Sup35 structural protein of [*PSI*^+^] [29,30]) aggregates via His6 affinity tag from yeast cells in the presence of SDS, followed by Rnq1-specific antibody staining [31]. The same approach was used later to prove that Rnq1 does not interact with the C-terminal domain of Sup35 [32].

### 2.3. Gel Filtration (Size Exclusion Chromatography) and Differential Centrifugation

Gel filtration allows for one to separate aggregated proteins from different tissues or cells. In this technique, the identification of proteins in the same fraction, their co-elution, is considered to be a result of their interaction. In the case of co-aggregation of the proteins, they are expected to be found together in fractions of high-molecular-weight complexes (Figure 1B). For example, the gel filtration of brain homogenates that were prepared from the AD transgenic mice followed by Western blot analysis has shown the presence of PrP and Aβ in the same fractions [33]. However, as the authors of this paper mentioned, the isolated aggregates may be found in the same fractions without direct interaction between them due to the similarity of their molecular weights or mobility. For this reason, the gel filtration assay is either used in combination with other methods and/or may be followed by immunoaffinity chromatography [34]. Analogous results could be obtained with differential centrifugation (Figure 1C). This technique was used to identify proteins that interact with Sup35 aggregates [35,36].

### 2.4. Colocalization in Cells and Tissues

Fluorescent microscopy is a widely used method to study the colocalization of proteins in the cells and investigate the molecular interaction between them. This approach requires labeling the proteins with different fluorescent tags. The most widely used fluorescent tag pairs are color variants of the green fluorescent protein such as CFP/YFP or RFP (dsRed, mCherry or mRFP1)/GFP. This approach is more suitable for unicellular organisms, such as yeast *Saccharomyces cerevisiae*, in which manipulation with plasmids containing fusion genes is a routine procedure. The colocalization experiments were employed to visualize the interaction of Sup35NM and Rnq1 aggregates during the de novo induction of [*PSI^+^*] prion [37,38]. The colocalization was also shown for Sup35NM-YFP with the following yeast chaperones: Ssa1, Ssa2, Sis1, Hsp104, and Hsp110 (Sse) labeled with CFP [39]. Another approach is to use fluorescent-labeled antibody staining. This method was used for co-immunolocalization of Aβ and PrP [24,40], Tau and α-Syn [28,41], Htt and TIA-1 [42], and AApoAII and AA fibrils [43].

Electron microscopy (EM) analysis is a more precise method of colocalization and could provide information about the orientation of separate protein aggregates relative to each other. For example, using immunogold EM, it was shown that the co-incubation of Tau and α-Syn leads to the formation of bundled fibrils labeled with both Tau and α-Syn antibodies. The analysis of these bundled fibrils reveals that they are composed of Tau or α-Syn aggregates (composed only by one protein) annealed “end-to-end” [41]. EM has also shown that pre-aggregated Aβ seeds aggregation of monomeric Tau in a cell-free assay [44].

The evidence of the colocalization between different amyloids that were obtained by this method cannot prove the direct interaction between their structural proteins. For this purpose, other techniques must be used, such as fluorescence cross-correlation spectroscopy (FCCS) or Förster resonance energy transfer (FRET). The FCCS is based on the monitoring of migration of differently labeled protein molecules, and their coordinated movement allows for researchers to suggest that molecules interact with each other. Another method, which is based on the analysis of fluorescence fluctuation, is FIDA (the fluorescent intensity distribution analysis technique). The FRET technique requires the application of two fluorescent tags with specific properties: emission spectrum of the first tag has to overlap with the excitation spectra of the second one. If such molecules are located in proximity to each other, the excitation of the first fluorophore will lead to the excitation of the second one. The detection of this energy transfer is the evidence of interaction between the analyzed molecules.

Indeed, advanced colocalization analysis together with FRET microscopy of brain sections stained for distinct protein aggregates demonstrated that several neurodegeneration-related proteins rarely, if at all, interact in human brain tissue [45]. The colocalization of Tau and α-Syn that had been found in the same cellular compartments [28] was confirmed by FRET [46,47]. The PrP sequences essential for the interaction with Aβ peptide were identified with the same approach [48]. The FCCS analysis revealed a strong interaction between yeast Sup35NM-GFP and Sis1-mCherry, as well as Hsp104-mCherry in the [*PSI*^+^] cells; interaction of Sup35 with other prion proteins, such as Ure2, Rnq1, or New1 was also shown by the same combination of the techniques [49]. Using the FIDA, it was shown that Tau and α-Syn can form co-oligomers and that co-aggregation happens even at nanomolar concentrations, but only in the presence of cationic aggregation inducers, such as Al^3+^ and Fe^3+^ or DMSO. Moreover, Tau phosphorylation by GSK3β strongly enhanced the formation of mixed oligomers [50].

### 2.5. Electrophoresis

A traditional biochemical approach, which was developed for the investigation of amyloid aggregates, like semi-denaturating detergent agarose gel electrophoresis [51,52], may be used for the investigation of protein co-aggregation. The formation of detergent-resistant aggregates of the protein only in cells with amyloid aggregates may be considered as evidence for co-aggregation. It was shown by the example of Pub1 protein, which aggregates only in the presence of amyloids of Sup35 or Rnq1 [53]. Also, changes in the size of amyloid aggregates upon overproduction or in the absence of another protein in the cell may allow for one to speculate that two proteins co-aggregate, but this strongly requires an additional proofs. For instance, the incorporation of Sfp1 into Sup35 aggregates was supposed based on such results supported by experiments demonstrating colocalization of these proteins [54].

### 2.6. Cross-Seeding

**Figure 2:**
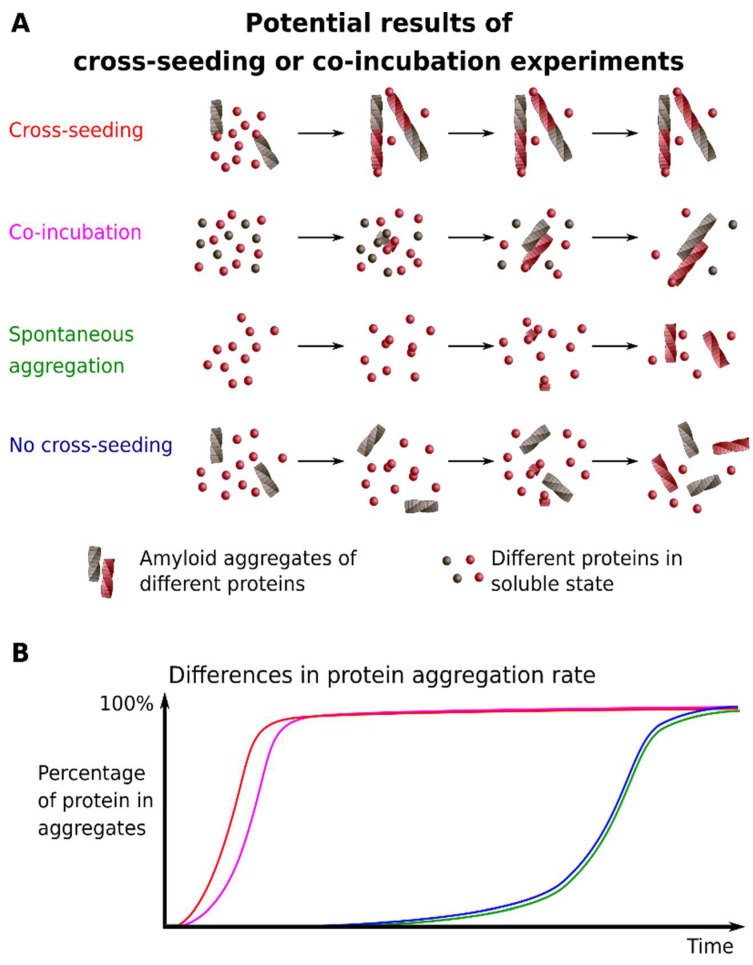
Schematic representation of cross-seeding and co-incubation. (**A**) The differences between molecular events upon cross-seeding or co-incubation of proteins; (**B**) The plot shows relative differences in the protein aggregation rate in cases shown on A. Colors on both panels correspond to each other.

Unlike the approaches that are listed above, the effective cross-seeding can provide data allowing for one to speculate that the soluble protein is incorporated into the pre-existing amyloid aggregates of another protein (Figure 2). This phenomenon, which is also called heterologous seeding, implies that preformed seeds (small aggregates or oligomers) of one protein accelerate the aggregation of other proteins (reviewed in [55,56]). The experimental design of a cross-seeding experiment is quite simple and assumes mixing the preformed fibrils and fresh solutions of the monomeric protein. A sample without added seeds serves as a control of spontaneous aggregation. A significant increase in aggregation rate induced by preformed fibrils indicates a possibility of cross-seeding. Different buffer systems and concentrations of preformed seeds drastically affect the efficiency of a cross-seeding [55,57].

**Figure 3:**
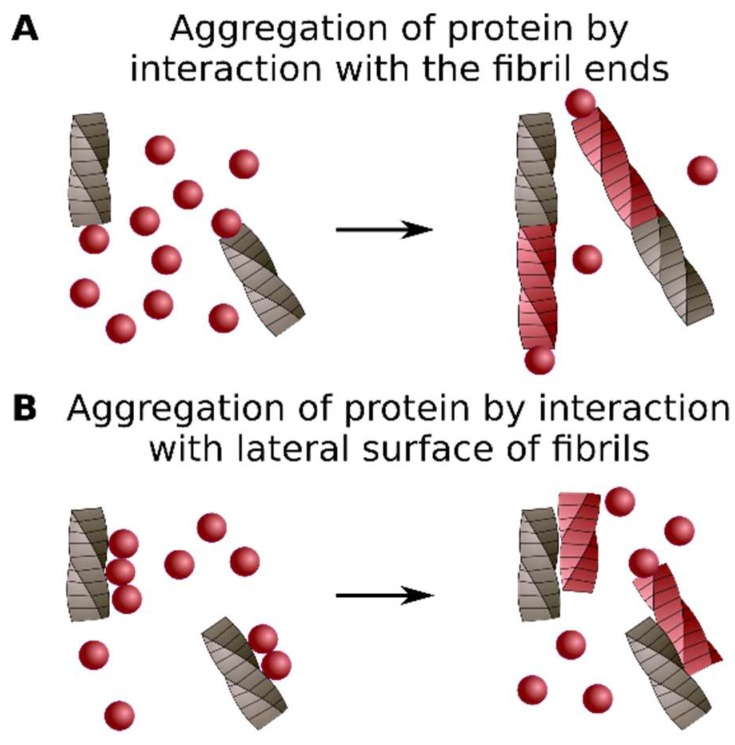
Cross-seeding mechanisms. Aggregation of the protein may be caused by interaction with fibril ends (**A**) or lateral sides (**B**). See details in the text.

One of the main limitations of the cross-seeding approach is the requirement for an in vitro system and pure protein samples. It is essential to highlight that formation of the aggregates of a specific protein by the addition of preformed aggregates may be linked to different molecular events. For example, monomers of the heterologous protein may stick to the ends of the fibrillar seeds, thus templating the structure of existing aggregates (Figure 3A) [37]. The preformed aggregates may also serve as a surface that adsorbs the heterologous protein. Consequently, a local increase in the protein concentration leads to de novo aggregation of the adsorbed protein without the incorporation into seeds (Figure 3B) [58]. These two examples of possible cross-seeding mechanisms, of course, do not cover all diversity of cases.

Different methods may be used for monitoring the aggregation kinetics. A number of such approaches are based on the aggregate staining with amyloid-specific dyes, like ThT or CR [59]. Also the size-exclusion chromatography, the transmission electron and the atomic force microscopy may be used [56,60]. ThT is used more frequently. This dye binds specifically to amyloid fibrils, leading to an increase in the fluorescent emission during the formation of amyloid fibrils [61]. Different modes of ThT interactions with amyloid aggregates were identified [62]. In several studies, the dynamic light scattering method was also used for the analysis of aggregation [63].

The critical step in the preparation of a monomeric protein for cross-seeding experiments is the removal of preformed aggregates [64]. Without this step, a detectable increase in the aggregation rate may be caused by the interaction of a monomeric protein with its own aggregates. The fibrils may be prepared by spontaneous assembly from disaggregated monomeric peptide or protein, each of which required customized protocols for fibril formation. Such protocols for Aβ peptides, polyQ peptide, human IAPP, and lysozyme are given in [65]. To normalize the molecular weights of fibrils, usually, a sonication step is used [64]. For example, in vitro cross-seeding was shown for Aβ and α-Syn [66], Aβ and Tau [44], IAPP, and α-Syn [60]. Another feature of these approaches is that they can examine chemically synthesized short protein fragments (for example, Aβ peptides) [64,66].

Cross-seeding experiments are widely used to characterize the interaction between the [*PIN^+^*] and [*PSI^+^*] prions in yeast. It was shown that aggregates of Rnq1 stimulated conversion of Sup35NM into the amyloid, although much less efficiently than Sup35NM stimulated its own conversion [37]. Rnq1 prion domain (Rnq1PrD) cross-seeded Sup35NM polymerization, and vice versa, both of the cross-seeded reactions had similar kinetic characteristics. Cross-seeding also occurs via the formation of hybrid aggregates between Sup35NM and Rnq1PrD revealed by EM [67]. Variant-specific [*PIN^+^*] fibers cross-seeded [*PSI^+^*] variants [68]. Also, Rnq1 seeds that were prepared from the full-length Rnq1 protein enhanced Sup35NM aggregation in vitro [32].

In some variations of cross-seeding experiments, one of the proteins under study can be obtained from the natural tissues, while another protein is obtained in vitro (reviewed in [56]). For example, PrP^Sc^ purified from the brains of scrapie-affected animals seeded synthetic Aβ [24]. Also, preformed fibrils of one protein may be used for the induction of aggregation of another protein in cultured cells, as it was done for α-Syn fibrils, which induced Tau aggregation in the cultured non-neuronal cells [69], or pre-aggregated Aβ seeds, which could facilitate Tau-aggregation in cell culture [44].

### 2.7. Co-Incubation of Monomeric Proteins

In this case, the interaction that starts with the two or more non-aggregated forms of proteins is studied (reviewed in [70], see Figure 2). For example, co-incubation of Tau and α-Syn induced aggregation of both proteins [41]. Also, the influence of varying concentrations of the full-length PrP^C^ (23–231) on the process of Aβ peptides oligomerization and fiber formation was shown [71]. In the same work, the effect of the PrP fragment (23–231 aa) on the preformed mature Aβ fibrils was investigated. The efficiency of Aβ peptides fibril formation in the presence of PrP was reduced, and the PrP fragments induced Aβ fibrils disassembly [71]. Co-aggregation of variants of IAPP was demonstrated by electrospray ionization-ion mobility spectrometry-mass spectrometry (ESI-IMS-MS) [72].

### 2.8. Proteomic Analysis of the Amyloid Interactome

A rapid development of modern proteomic techniques significantly improved the methodology for the analysis of protein co-aggregation with amyloids. Since the latest advances in this field are summarized in several reviews [73,74,75], here we present a few examples. Proteomic approaches can be classified into two groups: (i) methods for identification of proteins that comprise amyloid deposits and (ii) identification of proteins presented in amyloid-rich protein fractions.

The studies on the identification of proteins that are sequestered by pathological amyloids were the first attempts to apply proteomics to amyloid research [76,77]. Pathological amyloids typically form large deposits that can be detected by different histological techniques. A fragment containing an amyloid deposit can be isolated from formalin-fixed paraffin embedded tissue by laser capture microdissection (LCM). Proteins are extracted from the tissue specimen and are digested by trypsin. The resulting peptides are separated by high-performance liquid chromatography (HPLC) and identified by mass spectrometry (MS). Such a method provides efficient comparative analysis and allows for identifying dozens and hundreds of proteins that are sequestered by amyloid deposits [78,79]. However, it cannot resolve whether proteins co-aggregate with amyloid or non-specifically bind with it. LCM coupled with HPLC and MS can also be efficiently used in the clinical diagnostics of amyloidosis [80,81]. The limitation of the method is the size of amyloid deposits. They should be sufficiently large to be excised by LCM.

The second group of methods is based on the proteome-wide identification of proteins that are presented in amyloid-rich fractions. Such an approach was used to identify the proteins that are interacting with α-Syn [82,83], as well as phosphorylation-dependent α-Syn interactions [84]. The proteomic methodology was applied to find specific proteins that are enriched in AD hippocampal aggregates [85]. A quantitative proteomics approach was used to show that artificial β-sheet proteins forming amyloid-like fibrils sequester proteins enriched in intrinsically disordered (ID), or unstructured regions [86]. Consistent with this conclusion and using the similar approach, it was found that sets of proteins interacting with polyQ-expanded Htt were enriched for proteins with ID [87]. Also, model animals can be used to identify the proteins that are interacting with amyloids by a proteomic approach. For example, purified porcine brain synaptosomes were employed to investigate the interactome disease-related oligomeric α-Syn [88]. In the transgenic (Tg) mouse model of AD, several cytosolic proteins were identified that lose solubility during the accumulation of amyloids [89]. The sequestration of soluble proteins in aggregates during HD progression in a mouse was shown [90]. Proteomics approach in the *Caenorhabditis elegans* HD model was used to identify protein components of purified Q40-containing aggregates [91].

Different proteomic approaches were used to find proteins that are included in amyloid aggregates in yeast *S. cerevisiae*. The first of them allowed for the identification of different proteins that are associated with aggregates of the yeast prion [*PSI*^+^] [36]. Later, two proteome-wide methods allowing identification of amyloidogenic proteins were developed: TAPI (Technique for Amyloid Isolation and Purification) [92,93,94] and PSIA (Proteomic Screening and Identification of Amyloids) [95,96,97]. Both of the methods are useful for the identification of novel amyloids as well as proteins that co-aggregate with amyloids. For example, TAPI and PSIA revealed a set of proteins that co-aggregate with Htt (Htt103) aggregates [92,95]. The modified version of PSIA, including HPLC separation of tryptic peptides allowed for the rapid identification of protein determinants of the yeast [*NSI*^+^] prion [97] that for a long time have not been identified by traditional genetic approaches [98,99].

Overall, proteomic methods for the identification of amyloids and amyloid-associated proteins provide large data array of proteins that have potentially amyloidogenic properties or co-polymerize with amyloids. These data are extremely useful for the prediction of amyloid-amyloid interactions as well as interactions of amyloids with non-amyloid proteins. Nevertheless, false discovery rates remain an unresolved problem for proteomic data [100]; thus, each particular interaction needs to be individually validated by other methods.

### 2.9. Transgenic Animals

Various animal models, including rodents, non-human primates, *Danio rerio*, *Drosophila melanogaster*, and *C*. *elegans* are now developed to study amyloidogenesis (reviewed in [101,102,103,104,105,106,107,108]). All of these models have specific limitations, the most important of which is the incomplete similarity with the human pathology development.

There are many examples of mouse models that are used to study amyloid interactions. The generation of Tg mice with high levels of the human neuronal Aβ together with human α-Syn, followed by a comparison of their pathogenic effects alone and in combination allowed for one to suggest that interactions between α-Syn and Aβ are involved in the pathogenesis of LBD [109]. Double transgenic mice were employed to demonstrate the interaction between Tau and α-Syn [41]. Injection of pre-formed α-Syn fibrils assembled in vitro into the brains of Tg mice bearing human mutant Tau also was used to demonstrate cross-seeding from α-Syn to Tau in vivo [110]. In other experiments, the inoculation of mouse-adapted scrapie strains intracerebrally into Tg mice that overexpress human α-Syn [111] or injection of heterotypic Aβ-seeded Tau in Tg mice (TauP301S) [44] was applied to study the interactions between these proteins. The inoculation of mouse-adapted PrP^Sc^ into aged α-Syn Tg mice was used to prove that PrP^Sc^ could promote α-Syn pathology [112]. Non-vertebrate models, such as *C*. *elegans* and *D*. *melanogaster*, can be also used to study the amyloid-interacting proteins [113,114,115].

### 2.10. The Yeast S. cerevisiae as a Model System

Despite a traditional use of yeast system to study its prion networks, some aspects of mammalian proteins co-aggregation can also be studied while using this simple unicellular model (reviewed in: [116,117]). For example, in yeast system α-Syn and Tau interaction [118], as well as functional interactions between the voltage-dependent anion channel and α-Syn, were shown [119]. In a *S*. *cerevisiae* model of PD, a role of α-Syn in modulating sorting nexin 3 (Snx3)-retromer-mediated recycling of iron transporters was demonstrated [115]. A yeast-based prion nucleation assay has been developed. The investigated protein is considered as amyloidogenic if its fusion with the amyloid-forming domain of Sup35 leads to [*PSI*^+^] induction in the prion-free yeast cells [120].

Yeast *S*. *cerevisiae* is also used as a host in the yeast two-hybrid system (Y2H), which was specially developed to study interactions between non-yeast proteins. This system uses the reporter *lacZ* gene under the control of the *GAL1* promoter. The proteins of interest are fused with different parts of the Gal4 transcription factor. The interaction between these proteins restores functionality of Gal4p and leads to the *lacZ* expression, which, in turn, can be revealed by colony color on special media (reviewed in: [121,122,123]). This approach allows for the construction of interactomes for various amyloid proteins. For example, different Htt-interacting proteins were identified using Y2H screens [124,125]. However, not all of the proteins that were found in these screens were identical, which can be explained by the limitations of the method (reviewed in [125]).

### 2.11. Computational Approaches

A bioinformatic approach has shown the overrepresentation of proteins with α-helical coiled-coil regions in the interactomes of a subset of prions and disease amyloids [126]. Later, it was demonstrated that proteins interacting with polyQ proteins often contain coiled-coil regions and that enhancers of polyQ toxicity and aggregation are enriched in such regions [127]. Further analysis of polyQ proteins and their homologs revealed that the polyQ region usually had an exposed position that supports its involvement in protein-protein interactions [128]. The bioinformatic analysis of sets of prion, prionogenic, and prion-like proteins of *S*. *cerevisiae* allowed for the authors to identify specific interaction networks and to propose their role in gene regulation [129].

Several attempts have been made to use a systems approach to analyze all published data, including different PubMed datasets to construct the protein networks, called amyloid interactome, which reflects disease pathology. As a result, specialized interaction networks that are related to human amyloids were published [130,131].

Molecular dynamics simulations show that Aβ and α-Syn localized on a lipid bilayer surface are capable of forming ring-like hybrid structures that can make a pore in the membrane [26], hinting at the possibility of cross-dimerization between Aβ and α-Syn in the aqueous environment [132]. Thus, a bioinformatic prediction is efficiently used to reconstruct complex protein networks and to predict the structural and functional features of proteins interacting with amyloids.

### 2.12. Biophysical Approaches

The direct interactions between proteins (including monomers and fibers) can be monitored in real time using Surface Plasmon Resonance (SPR). In this case, one of the proteins under study is immobilized on a sensor chip, followed by the injection of the second protein over the chip. An increase in the resonance units that was observed after the injection shows an interaction between proteins. This approach was successfully used in several studies [133,134,135]. Another method, which can be applied to characterize the interaction between amyloids, is crosslinking. To prove that yeast Sup35 and Rnq1 physically interact and that Rnq1-Q298 and Sup35-N5 may represent an important site of contact, in vitro crosslinking was used [32]. Single molecular force spectroscopy measurements were also applied to measure specific interaction forces of curli protein CsgA to fibronectin [136].

### 2.13. Common Limitations of the In Vitro Approaches

Many of the approaches that are discussed require in vitro studies that have some limitations (reviewed in [56]). In particular, such experimental systems could not accurately reproduce a complex environment of living organisms. From another point of view, the concentrations of proteins analyzed may significantly exceed physiological amounts, which can provide misleading results. Also, most of in vitro experiments were usually done with the completely denatured proteins. Thus, it is essential to consider the results of in vitro approaches with the assumption of corresponding limitations. One example of the inconsistency of the results obtained in vitro and in vivo will be discussed in the next section. In particular, several proteins of stress granules were shown to form amyloid aggregates in vitro, but in vivo formation of these organelles is independent on amyloid aggregation (Section 3.3).

## 3. The Diversity of Amyloid Co-Aggregation Phenomenon

### 3.1. The Involvement of Protein Co-Aggregation in the Pathogenesis

Currently, several examples of interactions between amyloids and both amyloid-forming and monomeric soluble proteins are described. Some of them are likely to be associated with pathogenesis, while others are non-pathogenic or functional. Interactions between pathogenic amyloid-forming proteins are actively studied due to their possible involvement in the development of different, presumably neurodegenerative amyloidoses. Though such amyloidoses are associated with the aggregation of particular proteins, there are many cases of co-existence of different amyloids in single pathology. The AD is associated with the formation of extracellular Aβ plaques and intracellular neurofibrillary tangles that are formed by hyper-phosphorylated Tau peptide [137]. For a long time, these plaques and tangles have been considered to be spatially non-overlapping, but a recent Positron Emission Tomography study suggested that Aβ and Tau might form an interaction network in the brain [138]. Moreover, Aβ aggregates promote Tau hyperphosphorylation and aggregation in vitro [139]. The aggregates of α-Syn induce the formation of Tau fibrils in vitro, and both proteins induce the polymerization of each other in mouse model [41]. The aggregates of both mutant α-Syn and Tau were detected in the rare familial PD that is caused by the A53T α-Syn mutation [140]. Aβ and α-Syn oligomers cross-seeded the aggregation of each other in vitro [66]. Finally, a correlation between PD and AD was recently demonstrated [141], supporting the pathological role of interactions between α-Syn, Aβ, and Tau.

Another example of interactions between pathological amyloids is the interaction between PrP prion protein and Aβ. The normal isoform of the protein, PrP^C^, is a membrane-bound glycoprotein, the biological functions of which remain unclear [142]. Soluble PrP^C^ acts as the high-affinity receptor for Aβ oligomers [143] and this interaction activates signal transduction through metabotropic glutamate receptor, mGluR5 [144], to Fyn kinase, which hyper-phosphorylates Tau [145] that might trigger AD progression. The interaction between PrP^Sc^ and Aβ has been not fully investigated due to relatively rare cases of co-existence of PrP^Sc^ and Aβ deposits. Nevertheless, PrP^Sc^ deposits may co-distribute with Aβ plaques in the specific subtypes of sporadic [146] and familial [147] CJD, suggesting the possible role of this interaction in the pathogenesis.

#### 3.1.1. Interactions between Pathological Amyloids and QN-Rich Proteins

HD is caused by the expansion of Q-encoding repeats (36 to 180 glutamines) in the *HTT* gene, which includes the poly-Q containing exon-1 forming amyloid-like [148,149], presumably intranuclear inclusions [150]. The formation of poly-Q aggregates by Htt and several other proteins with expanded poly-Q repeats, like atrophin-1, is accompanied by significant changes in the transcription of pathogenesis-related genes. This effect is likely to be mediated by the sequestration of different proteins by poly-Q aggregates. The transcriptional co-activator CREB-binding protein (CBP) is sequestered by Htt and atrophin-1 aggregates, and this effect depends on the short poly-Q repeat in CBP [151]. Another transcriptional activator, Sp1, which binds to GC-rich elements in certain promoters, also binds mutant Htt, but only its soluble isoform [152]. Also, the aggregates of the Htt poly-Q containing exon-1 sequester the tumor suppressor protein p53 [153], transcriptional repressor protein mSin3a [154], TAFII130 transcriptional co-activator [155], TATA-binding protein (TBP) [149], and FUS RNA-binding protein [156]. Though the sequestration of all these proteins by poly-Q aggregates partially depletes their functional activity [151,153,155], and might thus be associated with pathogenesis, data are available that contradict this hypothesis [157]. A possible alternative explanation of the pathological transcriptional changes occurring in the presence of mutant poly-Q aggregates is direct transcriptional modulation, which was demonstrated for mutant Htt [158]. Overall, despite the fact that the molecular mechanism for the toxicity of poly-Q aggregates is still under the investigation, currently it is clear that the presence of such aggregates in the cell causes the sequestration of different potentially amyloidogenic proteins with Q- or/and N-rich regions. This effect was also revealed in the yeast model [94,95,159,160,161], suggesting that sequestration of QN-rich proteins by poly-Q (103 glutamines) aggregates is a general mechanism. The aggregates of several QN-rich yeast proteins (Def1, Pub1, Rpn10, Ent2, Sgt2, and Bmh2) acquire resistance to treatment with ionic detergents [94,162], thus, they are likely to co-aggregate with these aggregates. Also, poly-Q aggregates were shown to sequester preferably proteins with long ID regions [94]. Several proteins with long non-QN rich ID domains form meta-stable prions in yeast but are unrelated to amyloidogenesis [163]. Thus, sequestration of non-QN-rich ID-containing proteins by QN-rich amyloids might represent another important pathology-related mechanism.

#### 3.1.2. Interactions between Pathological Amyloids and Non-QN-Rich Proteins

Proteomic studies demonstrated that QN-rich amyloid aggregates also sequester proteins without QN-rich regions. For example, 54% of proteins co-aggregating with poly-Q (103) in the yeast model contain QN-rich regions, and only 7% of such proteins were detected among proteins co-aggregating with poly-Q in PC-12 cells [94]. Hundreds of proteins were shown to be co-purified with poly-Q aggregates from mouse brain, and a statistically significant enrichment with translation-related proteins was detected [164]. Another study revealed 747 proteins that were associated with poly-Q inclusions that were highly enriched with proteins involved in 14-3-3 signaling, microtubule-based transport, and proteostasis [165]. Moreover, the levels of production of more than 700 proteins in mouse brain were found to be dysregulated in the presence of inclusions that were formed by poly-Q with different lengths [166]. Thus, the repertoire of proteins co-aggregating with poly-Q is likely to to vary significantly, depending on the number of Q-repeats. Overall, protein sequestration in the case of poly-Q disorders is not limited to QN-rich proteins, but represents complex interaction network consisting of structurally and functionally distinct proteins, whose roles in pathogenesis are not fully elucidated.

The proteomics of AD revealed proteins co-aggregating with amyloid-β peptide not containing QN-rich regions. An early two-dimensional (2D)-gel electrophoresis study of LCM-isolated amyloid plaques detected 26 proteins [78]. A recent HPLC-MS proteomic research revealed 279 proteins present in amyloid plaques and demonstrated that plaques from patients with rapidly progressive AD were enriched with synaptic proteins, while the same from typical sporadic AD contained higher levels of neuronal proteins [108]. Interestingly, SDS-treated samples of amyloid plaques consist almost exclusively of Aβ [167], while samples that were treated with sarkosyl, which is a milder detergent than SDS, contain 11 proteins [168], most of which are known as the key AD-associated proteins: Tau, apolipoprotein E [169], serum amyloid P [170], and complement component 4 [171]. These data suggest that, in contrast to QN-rich amyloids that sequester relative large numbers of insoluble QN-rich amyloidogenic proteins, non-QN-rich amyloids, like Aβ, preferentially sequester non-amyloidogenic proteins that are not resistant to treatment with detergents. Since specific binding sites for these proteins have not been identified, we cannot discriminate whether these interactions represent sequestration or titration.

### 3.2. Yeast Prion Networks

The concept of prion and amyloid networks, proposing that an ensemble of amyloids and prions in the cell form an interacting sub-system, is actively studied in the models of yeast prions. Most of these factors represent QN-rich amyloids with parallel in-register cross-β structure. The initial example of such a network was found with the discovery of [*PIN*^+^], a prion of Rnq1 protein with an unknown function [172,173]. Although the biological role of [*PIN*^+^] remains mysterious, now it is clear that this prion acts as the heterologous spatial template initiating the induction of other prions in yeast, including QN-rich [174] and non-QN-rich prions, such as [*MOD*^+^] [175]. Though [*PIN*^+^] interacts with other prions at the initiation phase, it was shown that its aggregates do not physically interact with the aggregates of co-existing prions [68,176,177]. Some prions negatively affect the induction of each other. For example, [*PSI*^+^], inhibits [URE3] [30] formation [174,178], while [URE3] may inhibit [178] or induce [*PSI*^+^] appearance [173]. [*SWI*^+^] [179] enhances [*PIN*^+^] and [*PSI*^+^] appearance de novo, but it is destabilized when simultaneously present with [*PIN*^+^] and [*PSI*^+^] in a cell [180]. Moreover, it was clearly demonstrated that aggregates of Rnq1 colocalize with aggregates of Sup35 and Swi1 only at the initial steps of their aggregation; colocalization between mature aggregates was not observed [180]. Also, the interaction of prions may lead to appearance of some heritable traits. For example, [*PIN*^+^] increases translational read-through caused by [*SWI*^+^], but the aggregates of these prions do not colocalize [97]. Thus, according to the existing data, QN-rich prions physically interact rather at the induction phase, but when they co-exist in the cell, the antagonistic interactions are detected [97].

Similarly to the case of poly-Q amyloids, the yeast prions interact with a wide spectrum of proteins. For example, the overexpression of nucleoporin Nup100 in the [*NUP100*^+^] strains causes the co-aggregation of several QN-rich nucleoporins in yeast [181]. The Mss11, Sap30, and Msn1 proteins aggregate in [*SWI*^+^], but not in [*swi*^−^] strains when overexpressed [182]. On the other hand, a proteomic study, in which detergent-resistant fractions of [*SWI*^+^] strain were analyzed, did not reveal these proteins [97], suggesting that either their aggregates are likely to be non-amyloid without the overexpression or only little portions of these proteins form aggregates. Overall, it remains unclear whether QN-prions cause the co-aggregation of other QN-proteins at the endogenous level of expression, or QN-rich proteins co-aggregate with yeast prions only when overproduced.

Yeast prions also interact with different proteins that are considered to be non-amyloidogenic. The most detailed proteomic study of such interactions was performed in a [*PSI*^+^] model and revealed about 40 proteins, most of which were chaperones, stress-response and metabolic proteins [36]. Interaction of prions with Hsp104/70/40 chaperones is essential for prion propagation (reviewed in [17,183]), thus it is not surprising that these chaperones were detected in prion aggregates. Though there are pieces of evidence of direct physical interactions between prion-forming proteins and chaperones [184,185,186], the mechanisms of such interactions are poorly studied. According to the contemporary models, chaperones interact with amyloids through unstructured regions of the fibrils [187]. Hsp104 may interact with a fibril directly, as in the case of Sup35 amyloids [186], or with the aid of Hsp70 [188]. Several chaperones contain their own unstructured ID regions, and such regions may be important for the interactions with amyloids. For example, the ID region of Sgt2 chaperone, which is considered as a sensor for amyloid aggregates, is essential for interaction with poly-Q aggregates [94], while the C-terminal domain of Sgt2, which overlaps with the ID regions, is essential for an interaction with Sup35 [189]. Thus, unstructured ID regions may be important in the case of interaction between amyloid prions and soluble proteins.

### 3.3. Functional Amyloid Interactions

Previously, we discussed examples of proteins, interactions of which with amyloids have pathological or neutral consequences. In this section, we demonstrate that amyloid interactions may be beneficial. Recently, it was shown that human proteins Rip1 and Rip3 can form co-aggregates with amyloid properties, and this is a part of the signaling pathway triggering necrosis [190]. Further investigation of the necrosome assembly demonstrated that the formation of Rip1-Rip3 oligomers triggers Rip3 aggregation and autophosphorylation and it recruits mixed-lineage kinase domain-like protein (MLKL). It was proposed that Rip1-Rip3 amyloids play a role of a scaffold in the formation of necrosome, comprising a set of proteins [191]. This example of functional amyloid represents a case when two different proteins form heteroaggregate within the same structure. This hypothesis is supported by several pieces of evidence. First of all, capturing of the aggregates via Rip1-His6 demonstrated that both proteins, Rip1 and Rip3, are present in the eluate in equal amounts. The amyloid seeds comprising Rip3 effectively induce Rip1 aggregation. Also, it was shown that amyloid-forming regions in Rip1 and Rip3 include a conserved Rip homotypic interaction motif (RHIM) of corresponding proteins [190].

The analysis of the RHIM motifs diversity across different species allowed for us to propose their evolutionary link with the prion-forming domain of *Podospora anserina* proteins HET-s and HET-S [192]. These proteins are very similar [193] and implicated in heterokaryon incompatibility, and their co-aggregation is essential for this process [194]. HET-s in prion isoform forms infectious amyloid aggregates with β-solenoid structure [195,196], which refold soluble HET-S, so that it can bind and disturb lipid membranes, causing cell death. NMR data also let authors to suppose that two proteins adopt the same β-solenoid structure [194]. Further studies revealed another protein that was implicated in the cell death signaling. NWD2, Nod-like receptor, can form amyloid-like aggregates inducing the [Het-s] prion in a ligand depending manner. As HET-S, NWD2 can form a similar structure to Het-s one [197]. These data allowed to made it possible to suggest that NWD2/HET-S has another mechanism to trigger cell death in response to specific, but yet unknown, signal (reviewed in [198]).

The similarity of RHIM motifs assumes that they may adopt similar structures [192], though a recent study demonstrated the potential variety of these arrangements. Namely, the structure of the fibril formed by peptides from the amyloidogenic region of Rip1 and Rip3 was studied by the solid state NMR spectroscopy. According to these results, the fibrils contain two peptides in the cross-section, and Rip1 and Rip3 peptides alternate with each other along to fibril axis [199]. Nevertheless, it should be mentioned that the RHIM motif in Rip1 is flanked by two globular domains, which impose significant constraints on the fibril structure [200,201]. Therefore, a structural model of the amyloids that were formed by the full-length Rip1 and Rip3 proteins should consider that such fibril is surrounded by globular domains of the proteins from both sides. From this point of view, the alternative model is preferable for full-length Rip1 and Rip3 proteins: the amyloid core of the Rip1-Rip3 aggregate has a Het-s-like arrangement, but contains only one protein molecule in the fibril cross-sections, and different protein molecules stack one on the other [192,202].

Finally, cryptic RHIMs should be mentioned. They are found in *D*. *melanogaster* proteins, peptidoglycan recognition proteins (PGRP-LC and PGRP-LE), and Imd. Peptides with these motifs can form amyloids, and, as in the case with Rip1-Rip3, their aggregation is an essential step for signal transduction. Notably, the presence of preformed fibrils of PGRP-LE stimulates the aggregation of Imd [203]. RHIMs itself were found in many proteins implicated in necroptosis signaling [192,203,204,205,206], as well as in viral proteins [203,205,207,208,209]. Since several of these proteins inhibit necroptosis by preventing Rip1-Rip3 interactions [207], it was suggested that such proteins represent an example of a specific adaptation of pathogens to hosts [210].

Another well-known example of a functional amyloid cascade is the curli formation in bacteria. Proteins named CsgA-G (from curli-specific genes) are implicated in this process [211]. The major components of these structures are heteroaggregates of CsgA and CsgB [212]. Both proteins form amyloid aggregates [213,214]. Preformed aggregates of CsgB nucleate aggregation of CsgA [214,215]. Bacterial *csgB^−^* strains secrete soluble CsgA, which can be converted into curly by *csgB^+^* cells [211]. Taken together with the fact that CsgA and CsgB possess similar conserved amino acids motifs, these proteins are supposed to form the same arrangement within amyloid aggregates [214,216].

A number of different membrane-less organelles, including stress granules (SG), P-bodies, centrosome, Balbiani body, and Nucleolus were found in eukaryotic cells (reviewed in [217]). Among them, SG are the most studied complexes. These compartments include many proteins that can form hydrogels containing amyloid-like aggregates [218]. The major SG protein, Tia1, possesses several amyloid characteristics, can form protease and detergent-resistant fibrillar aggregates, and binds amyloid specific dyes [42,219,220,221]. Since SG were suggested to contain amyloid aggregates, proteins that are deposited in SG and are essential for their formation could be considered as co-aggregating with amyloids. Following this assumption, a number of proteins that are present in SG may be considered as examples of co-aggregation of proteins with amyloids. However, it has been shown recently that the formation of SG is not associated with amyloidogenesis [222]. Subsequent studies have demonstrated that the formation of membrane-less organelles does not require amyloids assembly and is rather connected with liquid-liquid phase separation (reviewed in [217]).

Recently discovered antimicrobial properties of several amyloids also may be considered as an example of the functional co-aggregation. In particular, it was shown that Aβ peptides behave as antimicrobial agents and inhibit the replication of several viruses. However, particular molecular mechanisms are still unknown (reviewed in [223]). Another potential example is eosinophil cationic protein which amyloid-like aggregation drives bacteria agglutination. This process may be triggered by the aggregation of surface-attached bacterial proteins and can result in the disruption of lipopolysaccharide bilayer and subsequent cell death [224].

## 4. Classification of Protein Co-Aggregation

**Figure 4:**
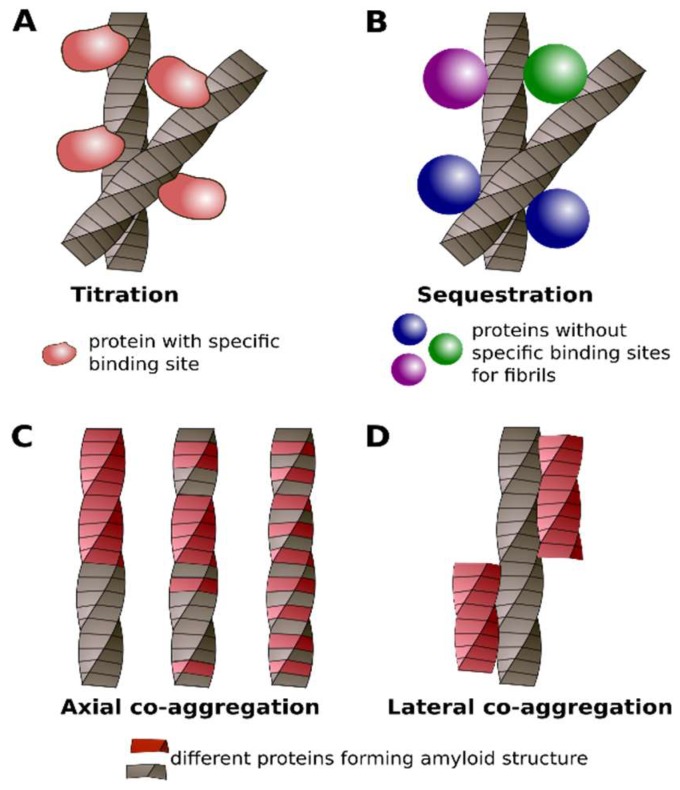
Different classes of protein co-aggregation with or via amyloids: titration (**A**), sequestration (**B**), axial (**C**) and lateral (**D**) co-aggregation. See details in the text.

In previous sections, we demonstrated that protein co-aggregation with amyloids is very versatile. It is apparent that the recent accumulation of data about different types of protein aggregation related to the amyloids requires their classification. Our analysis of these data resulted in the following classification in four classes: titration, sequestration, axial, and lateral co-aggregation. The first and second classes describe the interactions of monomeric proteins with amyloids. We propose the term titration for the interaction of certain proteins with amyloid aggregates via a specific binding site. It is the case when two proteins specifically interact with each other in the monomeric state and co-aggregate when one of them forms the amyloid (Figure 4A). One of such examples is a titration of yeast Sup45 by Sup35 aggregates [225]; in the native state, these proteins interact via specific regions [226,227]. We also propose to use this term to designate cases when “co-aggregating” proteins are the part of the same macromolecular complex and they might specifically interact through specific proteins.

In the second class of co-aggregation, which we propose to call sequestration, a particular protein can bind different types of amyloids. In this case, two proteins do not physically interact with each other in their monomeric states (Figure 4B). The well-known example of sequestration is the interaction of chaperones with protein aggregates. For example, yeast disaggregase Hsp104 is essential for fragmentation of most known yeast prions but generally does not interact with corresponding monomeric proteins via specific binding site [17,183] (Table 1). Although previously it was supposed that soluble M domain of Sup35 could interact with Hsp104 [185], we suggest that this interaction is rather non-specific. The interactions between ID-containing proteins, one of which is amyloidogenic, may also be classified as sequestration.

The next two classes describe interactions between amyloids that are formed by different proteins. In the third class, called axial co-aggregation, two (or more) proteins form a common fibril structure (Figure 4C). The three subclasses of such fibril heteroaggregates may be supposed: (i) two proteins alternate with each other along the fibril axis; (ii) proteins stack within fibril without any particular order; and, (iii) a part of the fibril is formed by the first protein and the other part is formed by the second protein (for instance, induction of aggregation of one protein by fibrils of another one) (Figure 4C). The examples of such interactions are Rip1 and Rip3, or Sup35 and Rnq1 proteins, respectively. From the methodological point of view, the verification of axial co-aggregation is one of the most challenging tasks. In this case, it is not sufficient to show that both proteins form amyloids. It is of importance that they colocalize or they are present in the same fraction of cell lysates. The proof that proteins are incorporated into one aggregate and form the same structure are required. These experimental difficulties explain the existence of only few examples of the well established axial co-aggregation (Table 1).

The fourth class, called lateral co-aggregation, comprises amyloids that interact with each other but do not form the same amyloid fibril. Such fibrils may stick to each other by their sides (Figure 4D). The interaction of Tau and α-Syn aggregates [41], or Aβ and Tau [58], could be considered as examples of this class of interactions. Numerous examples of cross-seeding between amyloidogenic proteins (Table 1) were described and can be related to either the third or fourth class of co-aggregation. However, in the most cases available data do not allow distinguishing between the axial and lateral types of co-aggregation. The summary of the differences between the four discussed classes of protein interactions with amyloids is presented in Table 2.

## 5. Conclusions

In this paper, we illustrated different aspects of protein interactions with amyloid aggregates and the impact of this process on pathogenesis or functional mechanisms. The review of the methods that are used in the field, its limitations and benefits demonstrated that an investigation of protein-amyloid interactions requires the combination of different cutting-edge approaches. The diversity of existing methods reflects the complexity of the amyloid interactomes and determines the boundaries that limit our ability to study this phenomenon. Nevertheless, recently developed methods allow for us to discriminate between different types of protein-amyloid interactions and propose their classification that includes four classes of interactions: titration, sequestration, axial, and lateral co-aggregation. We believe this first systematic classification of protein-amyloid interactions to encourage the investigators to add new examples of co-aggregating proteins to four proposed classes or to improve this classification if necessary.

## Figures and Tables

**Table 1:** Different examples of protein aggregation related to amyloids.

Amyloid-Forming Protein	Interacting Proteins	Class of Co-Aggregation	Experiments	References
Sup35	Ssa1, Ssa2, Hsp104, Sse1, Ssb1, Ssb2, Ydj1, Sis1	sequestration	differential centrifugation	[35,36]
	Sis1, Hsp104	sequestration	colocalization, FCCS	[49]
	Ssa1, Ssa2, Sis1, Hsp104, Hsp110 (Sse)	sequestration	colocalization	[39]
	Sgt2	sequestration	colocalization, differential centrifugation	[189]
	Ure2 ^1^, New1 ^1^	co-aggregation	colocalization, FCCS	[49]
	Rnq1 ^1^	axial co-aggregation	affinity chromatography, colocalization, seeding, crosslinking, FRET	[31,32,37,38,49,67,68]
	Sla2	titration	co-IP, differential centrifugation	[35,36]
	Sup45	titration	differential centrifugation	[225]
	Pub1^1^	co-aggregation	SDD-AGE	[53]
Rnq1	Pub1^1^	co-aggregation	SDD-AGE	[53]
Swi1	Mss1 ^1^, Sap30 ^1^, Msn1 ^1^	co-aggregation	colocalization	[182]
csgA	csgB ^1^	axial co-aggregation	seeding, SPR, structure modelling	[214,215,216]
	fibronectin	sequestration	single molecular force spectroscopy measurments	[136]
α-Syn	Tau ^1^	co-aggregation	seeding, colocalization, affinity chromatography, FRET	[25,28,41,50,69,110,118]
	Aβ ^1^	co-aggregation	seeding, co-IP	[26,66]
	IAPP	axial co-aggregation	seeding	[60]
Aβ	PrP ^1^	co-aggregation	co-IP, colocalization, seeding	[24,40,48,146,147]
	Tau ^1^	Lateral co-aggregation	seeding, colocalization, molecular dynamics simulations	[44,138,139]
AApoAII	AA ^1^	co-aggregation	seeding, colocalization	[43]
Htt, atrophin-1	CBP	sequestration	colocalization, co-IP	[151]
Htt	p53	sequestration	diffential centrifugation, colocalization	[153]
	mSin3a	sequestration	diffential centrifugation, colocalization	[154]
	TAFII130	sequestration	yeast two hybrid, co-IP	[155]
	TBP	sequestration	diffential centrifugation	[149]
	FUS	sequestration	colocalization	[156]
	Def1 ^1^, Pub1 ^1^, Rpn10 ^1^, Ent2 ^1^, Bmh2 ^1^	co-aggregation	PSIA	[94,162]
	TIA-1	sequestration	colocalization	[42]
Rip1/Rip3	Rip1 ^1^/Rip3 ^1^	axial co-aggregation	seeding, gel filtration, co-IP	[190]
	MLKL	titration	co-IP	[191]
HET-s	NWD2 ^1^	co-aggregation	seeding, colocalization	[197]
PGRP-LE	Imd ^1^	co-aggregation	seeding	[203]

^1^ The protein with supposed or known amyloidogenic properties.

**Table 2:** Differences between proposed classes of protein-amyloid interactions.

Titration	Sequestration	Axial Co-Aggregation	Lateral Co-Aggregation
The Interaction between Soluble Protein and Amyloid		The Interaction of Two Proteins in the Amyloid Conformation	
Soluble protein interacts with amyloid via specific binding site (s)	Soluble protein interacts with amyloid non-specifically	Molecules of different proteins stack along the fibril axis and form common amyloid fibril	Different proteins form separate amyloid fibrils which interact with lateral surfaces of each other

## Abbreviations

2D-GE	Two-dimensional gel electrophoresis
α-Syn	α-Synuclein
AD	Alzheimer’s disease
Aβ	Amyloid-β peptide
CBP	CREB-binding protein
CFP	Cyan fluorescent protein
CJD	Creutzfeldt Jakob disease
Co-IP	Co-immunoprecipitation
CR	Congo red
ESI-IMS-MS	Electrospray ionization-ion mobility spectrometry-mass spectrometry
FCCS	Fluorescence cross-correlation spectroscopy
FIDA	Fluorescent intensity distribution analysis technique
FRET	Förster resonance energy transfer
GFP	Green fluorescent protein
HD	Huntington’s disease
Htt	Huntingtin protein
*HTT*	Huntingtin gene
HPLC	High-performance liquid chromatography
IAPP	Islet amyloid polypeptide
ID	Intrinsically disordered (domains)
LBD	Lewy body disease
LCM	Laser capture microdissection
MLKL	Mixed-lineage kinase domain-like protein
MS	Mass-spectrometry
NMR	Nuclear magnetic resonance
PD	Parkinson’s disease
PrP	Prion protein: PrP^C^—normal isoform of the PrP: PrP^Sc^—prion isoform of PrP
PSIA	Proteomic screening and identification of amyloids
RFP	Red fluorescent protein
Rnq1PrD	Prion domain of Rnq1
Sarkosyl	Sodium lauroyl sarcosinate
SDS	Sodium dodecyl sulfate
SG	Stress granules
SPR	Surface plasmon resonance
Sup35NM	Amyloidogenic fragment of the Sup35, comprising N and M-domains of the protein
TAPI	Technique for amyloid purification and isolation
TBP	TATA-binding protein
TBP	TATA-binding protein
ThT	Thioflavin T
Tg	Transgenic animals
Y2H	Yeast two-hybrid system
YFP	Yellow fluorescent protein
